# Chemical Mechanical Polishing of Zerodur^®^ Using Silica and Ceria Nanoparticles: Toward Ultra-Smooth Optical Surfaces

**DOI:** 10.3390/nano15181391

**Published:** 2025-09-10

**Authors:** Houda Bellahsene, Saad Sene, Gautier Félix, Nicolas Fabregue, Michel Marcos, Arnaud Uhart, Jean-Charles Dupin, Erwan Oliviero, Joulia Larionova, Marc Ferrari, Yannick Guari

**Affiliations:** 1 Institut Charles Gerhardt Montpellier (ICGM), Centre National de la Recherche Scientifique (CNRS), École Nationale Supérieure de Chimie de Montpellier (ENSCM), University of Montpellier (UM), 34293 Montpellier, France; houda.bellahsene@etu.umontpellier.fr (H.B.); saad.sene@umontpellier.fr (S.S.); gautier.felix@umontpellier.fr (G.F.); nicolas.fabregue@umontpellier.fr (N.F.); erwan.oliviero@umontpellier.fr (E.O.); joulia.larionova@umontpellier.fr (J.L.); 2 Laboratoire d’Astrophysique de Marseille (LAM), Centre National de la Recherche Scientifique (CNRS), Centre National d’Etudes Spatiales (CNES) Aix Marseille University (AMU), 13013 Marseille, France; michel.marcos@lam.fr; 3 Institut des Sciences Analytiques et de Physico-Chimie pour l’Environnement et les Matériaux (IPREM), Centre National de la Recherche Scientifique (CNRS), Institut Mines-Télécom Mines Alès (IMT), Université de Pau et des Pays de l’Adour (UPPA), 64053 Pau, France; arnaud.uhart@univ-pau.fr (A.U.); jean-charles.dupin@univ-pau.fr (J.-C.D.)

**Keywords:** nanoparticles, chemical mechanical polishing, surface roughness, Zerodur^®^ mirror

## Abstract

This study investigates hyperpolishing of Zerodur^®^ substrates via chemical-mechanical polishing (CMP) using silica (SiO_2_) and ceria (CeO_2_) nanoparticles as controlled nano-abrasives. A pre-polishing stress-mirror stage was combined with systematic use of nanoparticles of variable size to evaluate surface-state evolution via optical rugosimeter, HRSEM, cross-sectional HRTEM, and XPS. A set of hexagonal mirrors with a circumscribed diameter of 30 mm was polished for one hour with each nanoparticle type. All tested slurries significantly improved surface quality, with both the smallest (37 nm) and largest (209 nm) SiO_2_ particles achieving similar final roughness, though larger particles showed a slight performance advantage that could be offset by longer polishing with smaller particles. CeO_2_ nanoparticles (30 nm) produced even better process efficiency and surface finishes than 37 nm SiO_2_, demonstrating higher chemical-mechanical polishing efficiency with CeO_2_. Sequential polishing strategies, first with 209 nm SiO_2_, then with 37 nm SiO_2_ and 30 nm CeO_2_, also enhanced surface quality, confirming trends from single-particle trials. One of the most effective protocols was adapted and scaled up to 135 mm Zerodur^®^ mirrors with spherical and plano geometries, representative of precision optical components. The strategic approach adopted to achieve a high-quality surface finish in a reduced processing time relies on the sequential use of nanoparticles acting as complementary nano-abrasives. Indeed, applying two hours of polishing with 209 nm SiO_2_ followed by two hours with 37 nm SiO_2_ yielded exceptional results, with area roughness (Sa) values of 1 Å for spherical and 0.9 Å for plano surfaces. These results demonstrate the capability of nanoparticle-assisted CMP to produce sub-nanometric surface finishes and offer a robust, scalable approach for high-end optical manufacturing.

## 1. Introduction

The vast majority of the more than 7500 known exoplanets have been detected by indirect means with radial velocity or transit methods [1]. On the other hand, direct-imaging optical systems are designed to detect exoplanets by capturing the faint light they emit near bright stars. In addition to relying on high-quality optical components, these instruments use adaptive optics and coronagraphic techniques to correct for atmospheric distortions and suppress starlight, making it easier to observe orbiting planets [2]. The amplitude of optical surface defects at spatial frequencies that adaptative optics cannot correct directly impacts the level of leakage around the coronagraph and, therefore, on the amplitude of residual image defects. If these residuals are too large, they mask the weak signal from the planet. For the coronagraphic instrument on NASA’s ROMAN Space Telescope mission (2027) [3], aiming for a contrast of 10^7^, i.e., a Jupiter-like planet 10 million times fainter than its host star, a surface form precision of 2 nm root mean square (rms) is required at medium and high spatial frequencies with an area roughness (Sa) lower than 5 Å. The challenge now is not simply to detect Jupiter-like exoplanets but to image Earth-like ones directly and characterize them in terms of radius, mass, and the composition of their atmosphere. Future missions dedicated to characterizing exoEarths like the Habitable World Observatory space mission will require contrast capabilities of the order of 10^9^ to 10^10^ and will, therefore, need even more precise optics, which implies a surface quality lower than 1 nm rms and an area roughness (Sa) lower than 1 Å [4].

Since the 1970s the Zerodur^®^ glass–ceramic, specially developed by Schott™ (SCHOTT France SAS, Colombes, France) for astronomical telescopes and instruments stability, has been one of the preferred classes of optical materials, due to its near-zero coefficient of thermal expansion and excellent homogeneity throughout the material. [5]. It consists of a rather complex composition of oxides, but is mainly formed of SiO_2_-Al_2_O_3_-Li_2_O [6], and exhibits a biphasic microstructure comprising a crystalline phase and an amorphous glassy matrix. The crystalline phase is composed of nanometric crystallites (a few tens of nanometers), which represents 70 to 78% of the entire material, uniformly embedded within the amorphous phase. In addition to its near-zero thermal expansion coefficient and good homogeneity, this material has very low or good surface finishing and high chemical stability. However, manufacturing Zerodur^®^ glass–ceramics using conventional milling or grinding techniques is challenging due to the significant difference in properties between the two components of the glass matrix: the amorphous body and the hard ceramic reinforcement (crystal particles). This disparity leads to the occurrence of surface defects, such as pits, cracks, and scratches, which prevent meeting the high precision optics requirements necessary for exoplanet detection and imaging [7,8,9].

To address the challenges encountered in the conventional machining for the planarization of Zerodur^®^ glass–ceramics, efforts have to be dedicated to the development of advanced polishing methods [10]. Chemical Mechanical Polishing (CMP) has become, for several decades, one of the preferred routes for eliminating topographic variations on different surfaces [11]. CMP is based on a three-component procedure including the surface material to polish, the polishing pad, and the polishing slurry. In a typical CMP process, the slurry is applied to the polishing pad and brought into contact with the material surface for polishing. Since both chemical and mechanical actions influence the effectiveness of CMP, and these actions are influenced by various factors, the CMP mechanism is complex and has been a subject of extensive research for many years [12,13]. In this process, the slurry plays an important role to obtain ultra-smooth and ultra-low damage surfaces [14,15]. Conventional CMP slurries typically comprise deionized water, abrasives, oxidizers, chelating agent, and pH regulators, and each of them plays a crucial role in determining the polishing effectiveness [16]. However, abrasives are the main component for material removal and, as such, have rightfully garnered significant attention from researchers. These abrasives are primarily made up of micrometric or submicrometric particles with various chemical compositions [16,17]. Among them, most studies have focused on micrometric particles of SiO_2_ [18], Al_2_O_3_, and CeO_2_ [19,20]. Thus, the use of 2.5 µm Al_2_O_3_ particles, by modifying the slurry dispersion, achieved optimum polishing quality for glass ceramic, resulting in a surface roughness of 3.16 nm rms [21]. Employment of colloidal solutions of submicrometric SiO_2_ or CeO_2_ particles (0.5 μm) improved the surface roughness of pre-polished Zerodur^®^, achieving up to 0.4 nm rms [22]. Furthermore, successively combining microparticles of varying compositions and sizes provided surface roughness reduction during the polishing of Zerodur^®^, as demonstrated by the sequential application of Al_2_O_3_ (10–12 µm) and CeO_2_ particles (1–3 µm) [23]. It has also been shown that infiltrating gallium followed by solidification into the surface microcracks of Zerodur^®^, prior to polishing with submicrometric CeO_2_ particles, allowed for the application of higher polishing pressures, reducing the polishing time by 21.4% while decreasing the surface roughness by a factor of 2.8 [6]. Notably, recent studies on silicon wafers as substrate focused on the use of nanoparticle solutions (i.e., particles smaller than 100 nm) as CMP slurries, such as SiO_2_ [24,25] or CeO_2_ [19] nanoparticles, which have demonstrated the ability to achieve surface roughness below 1 Å rms [24]. These findings collectively highlight the critical role of abrasive chemical composition, particle size (including in the nanometer range), and their sequential or combined application in pushing surface roughness below the angstrom level threshold.

In this paper, we investigate Zerodur^®^ surface hyperpolishing using SiO_2_ and/or CeO_2_ nanoparticles with controlled sizes as nano-abrasives for CMP, aiming to push the limits of this technique in achieving the surface roughness required for high-precision optics. Along with measuring surface roughness to compare the relative efficiency of these nanoparticles with different sizes and chemical composition, the pre-polished Zerodur^®^ surfaces were analyzed before and after hyperpolishing using various complementary techniques to try to gain insight into the surface chemistry. The successive combination of such nanoparticles with different sizes has allowed us to take a step forward to meet the challenge of reducing the surface roughness to Angstrom-level dimensions, constituting a notable advancement in this research area.

## 2. Materials and Methods

Tetraethoxysilane (TEOS) was purchased from ABCR (Karlsruhe, Germany), absolute ethanol was purchased from Merck (Darmstadt, Germany), ammonium hydroxides (25% and 30%) were purchased from Sigma-Aldrich (Steinheim, Germany), and Cerium (III) nitrate hexahydrate was purchased from thermo scientific (Waltham, MA, USA). Zerodur^®^ mirrors were purchased from TRIOPTICS France (Villeurbanne, France). Some of them were cut into a hexagonal shape (15 mm per side and 8 mm in thickness), while others were used as spherical mirrors with a diameter of 135 mm and a thickness of 17 mm, without further cutting.

### 2.1. Synthesis of Silica Oxide Nanoparticle

The dense silica nanoparticles were synthesized by using a previously reported method with slight modification [26].

**209 nm.** A total of 8 mL of ultrapure water and 400 mL of absolute ethanol were mixed together at 20 °C and then 24 mL (≈183 mmol) of ammonium hydroxide (30%) and 12 mL (≈53.8 mmol) of TEOS were added to the solution. A white suspension formed after the solutions were maintained at 20 °C in a thermostatic bath under stirring overnight. The nanoparticles were collected through centrifugation for 15 min at 37,565× *g* and washed one time with ethanol and three times with water before being stored in a buffer solution at pH 11. Infrared (IR, ATR): δ(Si–O–Si) = 452 cm^−1^, ν_s_(Si–O–Si) = 805 cm^−1^, ν_as_(Si–OH) = 954 cm^−1^, ν_as_(Si–O–Si) = 1060 cm^−1^; Zeta potential: −67.6 mV in water pH 11; Nitrogen adsorption: *S*_BET_ = 13 m^2^ g^−1^; *d*_TEM_ = 209 ± 11 nm, δ^29^Si (ss-NMR): −92.32 ppm (Q_2_) for 2.20%, −101.34 ppm (Q_3_) for 28.9%, −110.86 ppm (Q_4_) for 68.9%; and TGA: Estimated weight loss: 0–210 °C: 2.70%, 210–800 °C: 3.31%.

**53 nm.** Same protocol as for 200 nm SiO_2_ nanoparticles but with 200 mL of ultrapure water and 200 mL of absolute ethanol and temperature fixed at 30 °C. Infrared (IR, ATR): δ(Si–O–Si) = 454 cm^−1^, ν_s_(Si–O–Si) = 810 cm^−1^,ν_as_(Si–OH) = 956 cm^−1^, ν_as_(Si–O–Si) = 1072 cm^−1^; Zeta potential: −38.5 mV in water pH 11; Nitrogen adsorption: *S*_BET_ = 62 m^2^ g^−1^; *d*_TEM_ = 53 ± 6 nm, δ^29^Si (ss-NMR): −91.85 ppm (Q_2_) for 1.5%, −101.08 ppm (Q_3_) for 34.3%, −110.81 ppm (Q_4_) for 64.2%; and TGA: Estimated weight loss: 0–100 °C: 2.92%, 200–450 °C: 1.31%, 450–600 °C: 1.38%, 600–800 °C: 0.78%.

**37 nm.** Same protocol as for 200 nm SiO_2_ nanoparticles but using 24 mL (≈156 mmol) of ammonium hydroxide (25%) and increasing the temperature to 75 °C. Infrared (IR, ATR): δ(Si–O–Si) = 450 cm^−1^, ν_s_(Si–O–Si) = 800 cm^−1^,ν_as_(Si–OH) = 948 cm^−1^, ν_as_(Si–O–Si) = 1065 cm^−1^; Zeta potential: −32.4 mV in water pH 11; Nitrogen adsorption: *S*_BET_ = 107 m^2^ g^−1^, *d*_TEM_ = 37 ± 5 nm, δ^29^Si (ss-NMR): −91.85 ppm (Q_2_) for 2.8%, −100.97 ppm (Q_3_) for 30.4%, −111.10 ppm (Q_4_) for 66.8%; and TGA: Estimated weight loss: 0–200 °C: 2.76%, 200–460 °C: 2.34%, 450–800 °C: 1.00%.

### 2.2. Synthesis of Cerium Oxide Nanoparticle

Cerium oxide nanoparticles of two distinct sizes were synthesized using separate methods: 17 nm particles were prepared via a coprecipitation protocol adapted from K. Sakthiraj [27], while 30 nm particles were obtained through a hydrothermal procedure based on the approach developed by Wang et al. [28]

**30 nm.** A total of 5.6 g (≈12.9 mmol) of cerium (III) nitrate hexahydrate was dissolved in 21.8 mL of ultrapure water with 3.2 mL of ammonium hydroxide (30%). After reaction for 24 h in a closed container at 250 °C, the CeO_2_ NPs were centrifuged during 10 min at 37,565× *g* and washed three times with water and ethanol and stored in an acidic solution at pH 4.5 to prevent any aggregation. Infrared (IR, ATR): δ(O–Ce–O) = 368 and 380 cm^−1^, ν(Ce–O) = 418 cm^−1^, δ(H–O–H) = 1552 cm^−1^ (adsorbed water), ν(OH) = 3565 cm^−1^ (surface hydroxyl groups and adsorbed water); TGA: Estimated weight loss: 0–150 °C: 1.08%, 150–290 °C: 3.56%, 290–500 °C: 1.20%, 500–800 °C: 0.33%; Nitrogen adsorption: *S*_BET_ = 33 m^2^ g^−1^, *d*_TEM_ = 30 ± 5 nm. XRD: Space group: Fm3m, space group number: 225, a = b = c = 5.41 Å. XPS (Ce3d): Presence of both Ce^3+^ and Ce^4+^ species with a Ce^3+^/Ce^4+^ ratio of ~0.38; Ce^4+^ shake-up satellite peak observed at 917 eV.

**17 nm.** A total of 15.0 g of cerium (III) nitrate hexahydrate was dissolved in 345 mL of water at 60 °C. Ammonium hydroxide (25%) was added dropwise until a pH of 9.5 (≈around 12 mL). The solution was heated for 2 h, until it appeared milky. The nanoparticles were washed twice with water using a centrifuge during 10 min at 37,565× *g*. They were stored in water with a pH of the solution measured at 4.5. Infrared (IR, ATR): δ(O–Ce–O) = 364 and 373 cm^−1^, ν(Ce–O) = 410 cm^−1^, δ(H–O–H) = 1540 cm^−1^ (adsorbed water), ν(OH) = 3552 cm^−1^ (surface hydroxyl groups and adsorbed water); TGA: Estimated weight loss: 0–150 °C: 1.39%, 150–290 °C: 3.72%, 290–800 °C: 2.11%; Nitrogen adsorption *S*_BET_ = 49 m^2^ g^−1^; *d*_TEM_ = 17 ± 6 nm. XRD: Space group: Fm3m, space group number: 225, a = b = c = 5.41 Å. XPS (Ce3d): Presence of both Ce^3+^ and Ce^4+^ species with a Ce^3+^/Ce^4+^ ratio of ~0.4; Ce^4+^ shake-up satellite peak observed at 917 eV.

### 2.3. Pretreatment Polishing of Zerodur^®^ Mirrors

The Zerodur^®^ substrates investigated in this study comprised two categories: (*i*) small-scale hexagonal mirrors with a circumscribed diameter of 30 mm used for preliminary process validation; and (*ii*) large-scale spherical mirrors of 135 mm representative of real-world applications, including one plano mirror and one with a spherical profile. Both categories underwent an identical, multi-step pre-treatment protocol aimed at establishing the required surface form (spherical or planar) and achieving the necessary surface quality for subsequent nanoparticle-assisted hyperpolishing. Initially, coarse-grain abrasives were employed to remove several millimeters of bulk material from the substrate, shaping the overall geometry. For the spherical mirrors, this step included the controlled generation of curvature, while the plano mirror was simultaneously maintained to strict flatness specifications. A reuniting step followed, wherein the mirror and tool surfaces were matched using 250 μm grade abrasives to refine the surface form and ensure an even material interface. Subsequently, a softening phase was conducted to minimize residual surface roughness and prepare the substrates for fine polishing. Abrasive grit sizes were gradually reduced from 100 μm to 5 μm, with meticulous cleaning between each step to eliminate cross-contamination and prevent the introduction of micro-defects. By the end of this phase, the mirror surfaces, whether plano or spherical, exhibited a uniform satin texture with minimal micro-scratches or pits, providing an optimal base for the final hyperpolishing stage. For the small hexagonal mirrors, polishing operations during this stage were conducted in a staggered configuration, using blocks of seven elements. This configuration allowed for efficient material removal and homogeneous treatment over multiple units, facilitating comparative analysis and validating the repeatability of the process on segmented optics. This unified pre-treatment approach successfully demonstrated its adaptability across both mirror categories, ensuring precise form control and low surface roughness, regardless of substrate size or geometry. The hyperpolishing procedure was carried out using nanoparticle slurries at 25 wt% in a buffer solution (pH = 11) and a metal pad coated with a CHEMPOL cloth made of alveolate polyurethane with open porosity.

### 2.4. Characterizations

Transmission electron microscopy (TEM) images of nanoparticles were recorded at 100 kV with a JEOL 1400 Flash (Tokyo, Japan). Nanoparticles’ samples for TEM measurements were deposited from suspensions on copper grids and allowed to dry before observation. The size distribution histograms were determined using enlarged TEM micrographs taken at a magnification of 100 K on a statistical sample of ca. 100 nanoparticles. Thin lamellae of Zerodur^®^ mirrors were prepared using a FEI Helios 600 Nanolab Dual Beam system (Hillsboro, OR, USA) equipped with electron and Ga^+^ ion columns. Regions of interest were identified via scanning electron microscopy (ZEISS Gemini SEM 500, Carl Zeiss AG, OBerkochen, Germany), then locally protected with a thin carbon layer followed by a platinum deposition. Milling was performed using a focused Ga^+^ ion beam accelerated at 30 kV and incident at 52°. Two stepped trenches were etched on either side of the lamella to a depth of ~5 µm. Thinning was carried out by alternating material removal from both sides, progressively reducing the beam current and voltage for final polishing. The lamella was then extracted and transferred in situ onto a TEM grid using a micromanipulator. High-resolution cross-sectional transmission electron microscopy (HRTEM) measurements were performed on a JEOL 2200 FS instrument (Tokyo, Japan). High-resolution scanning electron microscopy (HRSEM) was carried out on a Hitachi S-4800 microscope (Tokyo, Japan). Dynamic light scattering (DLS) measurements were performed using a Malvern Zetasizer Nano ZEN3600 (Worcestershire, UK) to determine the nanoparticles’ hydrodynamic diameter. Zeta potential measurements were carried out using a Zetasizer Nano ZS Model ZEN3600 (Worcestershire, UK) equipped with a DTS1070Zetacell (Malvern, Worcestershire, UK), in water at pH 11 for SiO_2_ and 4.5 for CeO_2_ at 25 °C, with an equilibration time of 120 s and automatic measurement settings. The data were treated with Zetasizer software version 7.03 using a Smoluchowski model. Thermogravimetric analysis (TGA) was performed using a Netzch STA 409 PC analyzer (Selb, Germany) in the 20–800 °C temperature range at a heating speed of 5 °C min^−1^ under argon. XRD diffractograms were recorded with the PANalytical X’pert diffractometers in powder configuration (Worcestershire, UK). The diffractometer is equipped with a Cu anode (1.5418 Å). The measures are made at room temperature between 10° and 85° in 2θ with a step of 0.0033° and a time of 185 s per step. FTIR spectra were recorded using a PerkinElmer Spectrum two spectrophotometer (Waltham, MA, USA); all samples were dried under vacuum prior to analysis to remove residual water. The specific surface area (*S*_BET_) was calculated according to the Brunauer–Emmett–Teller (BET) method. Prior to measurement, the samples were dried under vacuum at 80 °C to remove any residual moisture. All solid-state NMR experiments were performed on a Varian VNMRS 300 MHz (7.05 T) NMR spectrometer (Santa Clara, CA, USA). A 7.5 mm Varian T3 HXY magic angle spinning (MAS) probe was used for ^29^Si experiments, at operating frequency of 564.33 MHz. One Pulse ^29^Si MAS NMR spectra were recorded spinning at 5 kHz, with ^1^H decoupling during acquisition. A recycle delay of 60 s was used and total of 4000 scans were recorded for each sample. The surface roughness was measured with a Wyko NT9000 Series optical systems (Veeco Instruments Inc., Plainview, NY, USA). Field of view and spatial sampling are based on full resolution 640 × 480 pixels measurement array, 9.9 μm × 9.9 μm pixel size. Surface roughness parameters including Sa (average roughness), Sq (root mean square roughness), Pa (peak-to-valley height), and Pq (root mean square height) were extracted using Mountains Map^®^ Expert 9.2.10042 software. X-Ray photoelectron spectrometry (XPS) experiments on the different nanomaterials were achieved with a Thermo K-alpha spectrometer (Waltham, MA, USA) equipped with a hemispherical analyzer and a micro-focused (400 µm diameter microspot) monochromatic radiation (Al Kα, 1486.6 eV) operating under a residual pressure of 3 × 10^−9^ mbar. Pass energy was adjusted to 40 eV. To compensate the charge effects occurring during the analysis, a dual beam charge neutralization system (low energy electrons and Ar^+^ ions), which has the unique ability to provide consistent charge compensation, was used. The calibration of all spectra was based on the binding energy of carbon 1s orbital at 285.0 eV. The mathematical fitting was performed with the software Casa XPS version 2.3.15 using a least-squares algorithm and a non-linear baseline (Shirley). The experimental curves peaks were fitted using a combination of Gaussian (70%) and Lorentzian (30%) distributions.

## 3. Results and Discussion

### 3.1. Synthesis and Characterizations

#### 3.1.1. SiO_2_ Nanoparticles

**Figure 1:**
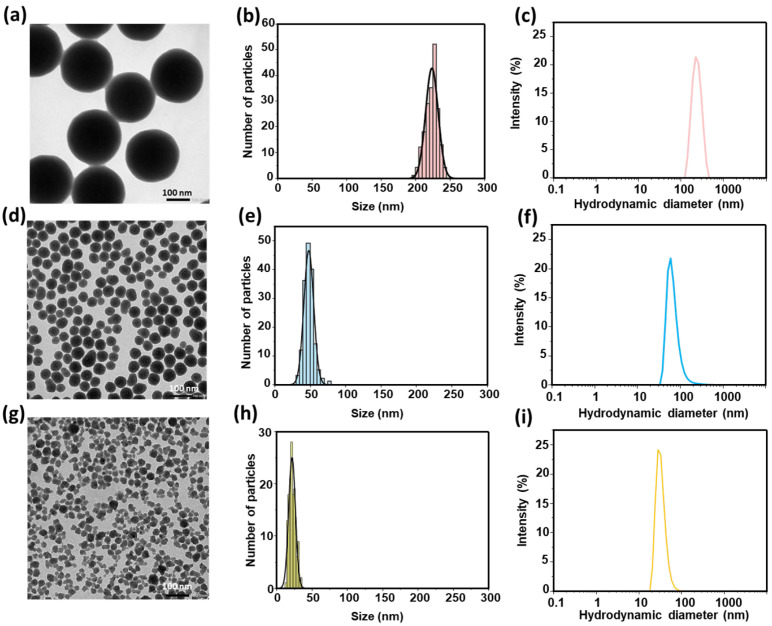
Silica nanoparticles with average diameters of 209 nm (**a**–**c**), 53 nm (**d**–**f**), and 37 nm (**g**–**i**). For each size, TEM images are shown in (**a**,**d**,**g**), particle size histograms from TEM analysis in (**b**,**e**,**h**), and hydrodynamic diameter distributions from DLS measurements in (**c**,**f**,**i**). Scale bars = 100 nm.

The synthesis of silica nanoparticles was based on the controlled hydrolysis and condensation of tetraethoxysilane (TEOS) following the Stöber method [29]. By systematically varying reaction parameters (Table 1), namely temperature, H_2_O/ethanol volume ratio, and catalyst concentration [30,31,32], it was possible to tune the average particle diameter across three target sizes (30, 50, and 200 nm). Transmission electron microscopy (TEM) was employed to assess particle morphology and size distribution. Representative micrographs (Figure 1) confirm that all three batches exhibit a highly uniform, spherical morphology and narrow size distributions. Statistical analysis of over 100 particles per sample yielded mean diameter 37 ± 5 nm, 53 ± 6 nm, and 209 ± 11 nm, respectively (Figure 1a,d,g). Dynamic light scattering (DLS) measurements further confirmed the presence of a distinct population for each nanoparticle size, with no aggregation observed in the hydrodynamic size profiles (Figure 1c,f,i). The agreement between TEM and DLS data indicates that the particles remain well-dispersed under the measurement conditions. Overall, these results validate the robustness of the hydrolysis–condensation method for producing size-tunable, non-aggregated silica nanoparticles. The synthesis of spherical SiO_2_ particles with well-controlled diameters was achieved reproducibly, yielding particles with low size dispersion and in quantities adequate for further applications. This criterion is critical for our polishing trials, which aim to investigate size-dependent effects and, therefore, require nanoparticles with well-defined sizes, where robust colloidal stability and precise control of nanoparticle size and morphology should be essential to achieve surface roughness on the order of angstroms.

**Figure 2:**
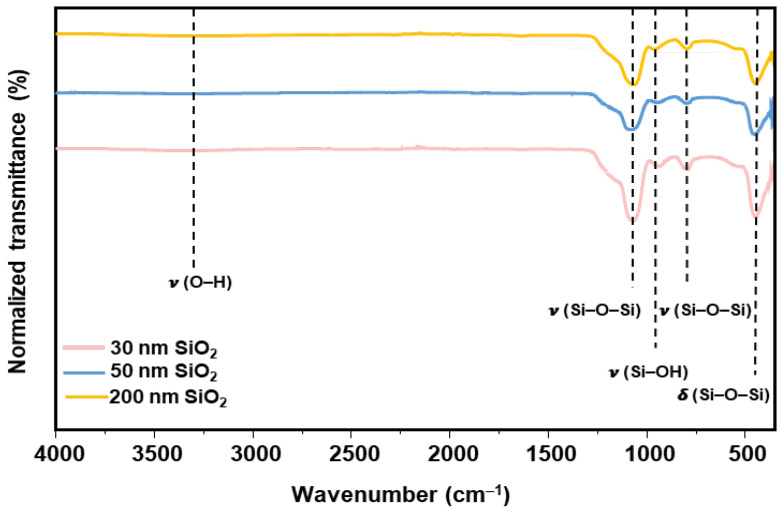
FTIR spectrum of the synthesized SiO_2_ nanoparticles, displaying the characteristic vibrational bands of silica.

Fourier transform infrared (FTIR) spectra of the as-synthesized SiO_2_ nanoparticles in Figure 2 reveal four principal bands characteristic of amorphous silica. The strong band at around 1060 cm^−1^ corresponds to the asymmetric stretching bonds, ν_as_(Si–O–Si), and the one at 952 cm^−1^ indicates the presence of O–H stretching bonds [33,34]. The band at 805 cm^−1^ is attributed to the symmetric Si–O–Si stretching mode ν_s_(Si–O–Si), and the low-frequency band at 452 cm^−1^ arises from the Si–O–Si bending vibration δ(Si–O–Si). These assignments are in agreement with values established in the literature for amorphous silica, confirming both the chemical composition and the absence of solvent or adsorbed residues in our samples [35,36].

**Figure 3:**
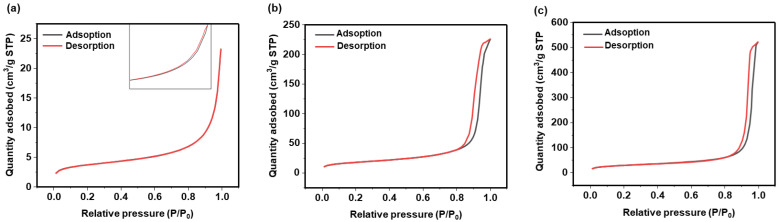
Nitrogen Adsorption and Desorption isotherms of SiO_2_ NPs of the following: (**a**) 209 nm; (**b**) 53 nm; and (**c**) 37 nm. The adsorbed nitrogen volume is given in cm^3^/g under standard temperature and pressure (STP) conditions. The inset in (**a**) presents a magnified view in the high relative pressure region, revealing the presence of a hysteresis loop.

The nanoparticles exhibit a non-porous structure as confirmed through nitrogen adsorption analysis (Figure 3) exhibiting isotherms of Type II for the three sizes of SiO_2_ nanoparticles. According to the literature, Type II isotherm suggests that the silica oxide nanoparticles are either non-porous or possesses interparticle porosity (pores with diameters > 50 nm). This is further confirmed by the hysteresis loop, which is not well-pronounced and exhibits an H3-type configuration. This is a characteristic more commonly associated with this type of isotherm [37]. The initial concave region reflects the formation of a monolayer, while the steep rise at higher pressures indicates unrestricted multilayer adsorption [38]. The obtained specific surface areas, *S*_BET_, are equal to 13, 64 for and 107 m^2^ g^−1^ for, respectively, the SiO_2_ nanoparticles of 209, 53, and 37 nm. These results are consistent with dense silica nanoparticles exhibiting a surface area associated with interparticle porosity.

**Figure 4:**
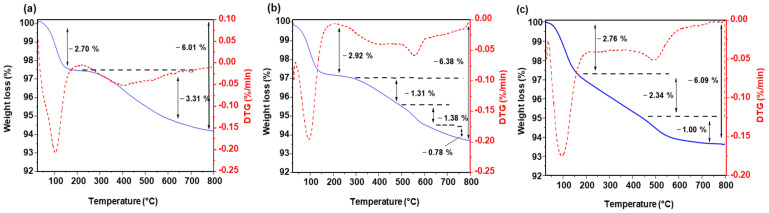
TGA (solid blue) and derivative thermogravimetric (DTG) (dashed red) curves for SiO_2_ nanoparticles of different sizes: (**a**) 209 nm, (**b**) 53 nm, and (**c**) 37 nm.

The thermogravimetric analysis (TG) curves (blue) reveal a multi-step mass loss process, while the derivative thermogravimetric (DTG) curves (red) highlight the decomposition stages at corresponding temperature ranges (Figure 4). The initial weight loss observed for all samples below 150 °C can be attributed to the removal of physically adsorbed water molecules and residual ammonia, as described by Jafarzadeh et al. [39]. Between 150 and 300 °C, the weight loss corresponds to the loss of trapped water, ammonia, and ethanol molecules. Between 300 and 800 °C, the weight loss is primarily due to the dehydroxylation of residual Si–OH groups and the degradation of the remaining SiOEt species. However, FTIR analysis (Figure 2) confirms the absence of characteristic C–H bands, indicating that the organic groups are absent and that the observed weight loss is mainly associated with the dehydroxylation of the Si–OH species. This overall weight loss is comparable across the three samples, with values of 3.31, 3.47, and 3.34%, respectively, for nanoparticles of 209 nm, 53 nm, and 37 nm. Note that the weight loss begins earlier for the smallest nanoparticles’ sample.

**Figure 5:**
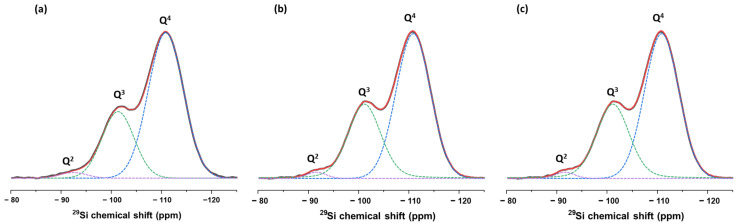
Solid state NMR spectra of SiO_2_ nanoparticles of the following: (**a**) 209 nm; (**b**) 53 nm; and (**c**) 37 nm.

Figure 5 presents the ^29^Si solid-state nuclear magnetic resonance (SSNMR) spectra of the synthesized silica nanoparticles, revealing three distinct resonances centered at approximately −111, −101, and −91 ppm. Spectral deconvolution reveals that the predominant signal corresponds to the resonance at −111 ppm, associated with Q^4^ silicon sites (fully condensed Si(OSi)_4_ environments), characteristic of a highly crosslinked silicate network. This Q^4^ contribution accounts for 68.9% in 209 nm, 64.2% in 53 nm, and 66.8% in 37 nm SiO_2_ nanoparticles. The resonance at −101 ppm is attributed to Q^3^ sites, corresponding to Si(OSi)_3_(OH) environments, which represent partially condensed silicon species containing one non-bridging oxygen. The Q^3^ signal represents 28.9, 34.3, and 30.4% of the total area for 209 nm, 53 nm, and 37 nm SiO_2_ nanoparticles, respectively. A minor peak near −91 ppm corresponds to Q^2^ environments of Si(OSi)_2_(OH)_2_, which is indicative of more weakly condensed silicate units with two non-bridging oxygens. It contributes 2.20, 1.50, and 1.80% for 209, 53, and 37 nm nanoparticles, respectively. Based on the above-described characterizations including FTIR and TGA, the Q^3^ and Q^2^ signal can be attributed to Si-OH groups, as we have no evidence of SiOEt species presence. These results demonstrate the silicate structure of the SiO_2_ nanoparticles with same condensation degree calculated from the relative intensities of Q^2^, Q^3^, and Q^4^ of ca. 91% [40].

#### 3.1.2. CeO_2_ Nanoparticles

**Figure 6:**
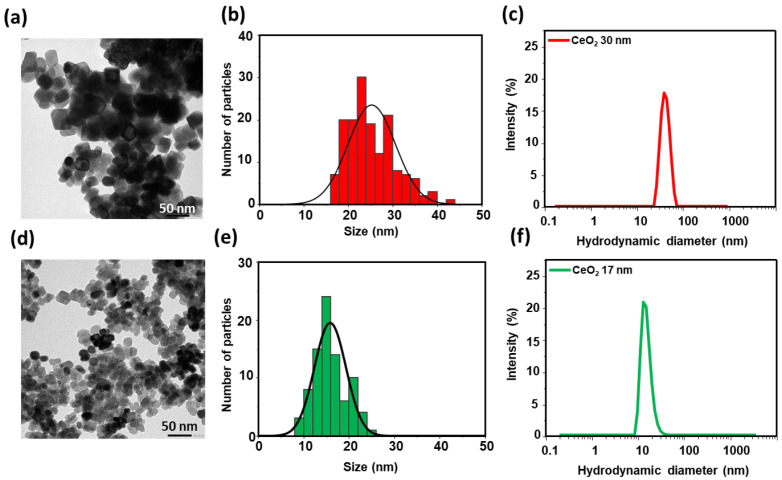
Ceria nanoparticles with average diameters of 30 nm (**a**–**c**) and 17 nm (**d**–**f**). For each size, TEM images are shown in (**a**,**d**), particle size histograms from TEM analysis in (**b**,**e**), and hydrodynamic diameter distributions from DLS measurements in (**c**,**f**). Scale bars: 50 nm.

The preparation of ceria nanoparticles of different size was based on a coprecipitation method [27] for targeted 15 nm nanoparticles and through a hydrothermal procedure [28] for the ones of 30 nm. TEM was employed to evaluate their morphology and size distribution. Representative micrographs shown in Figure 6 reveal that both samples are highly uniform. Statistical analysis of over 50 particles per sample indicates mean diameters of 17 ± 6 nm for the small-sized and 30 ± 5 nm for the larger ceria nanoparticles. The close correspondence between target and measured sizes underscores the effectiveness of the synthesis protocols in achieving precise control over nanoparticle dimensions with both coprecipitation and hydrothermal methods.

**Figure 7:**
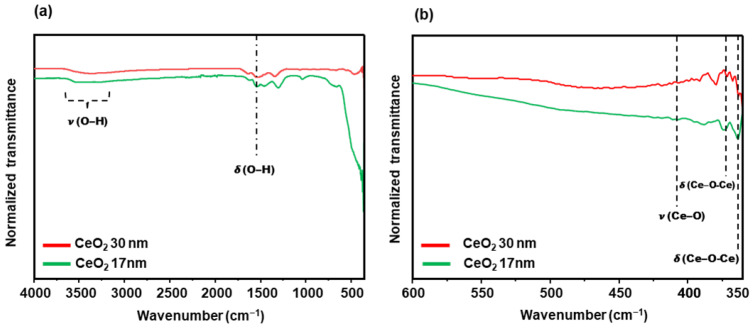
(**a**) FTIR spectra of Cerium oxide nanoparticles; (**b**) Magnification of the spectra in the 600–350 cm^−1^ window.

As illustrated in Figure 7, the FTIR spectrum of the as-synthesized ceria nanoparticles shows a broad O–H stretching band around 3550 cm^−1^, which can be attributed to both surface hydroxyl groups and adsorbed water. A bending mode of adsorbed water is also observed at 1540 cm^−1^. The Ce–O lattice stretching vibration appears at 410 cm^−1^, along with two closely spaced Ce–O–Ce bending modes at 370 and 363 cm^−1^, typical of the fluorite structure of CeO_2_.

**Figure 8:**
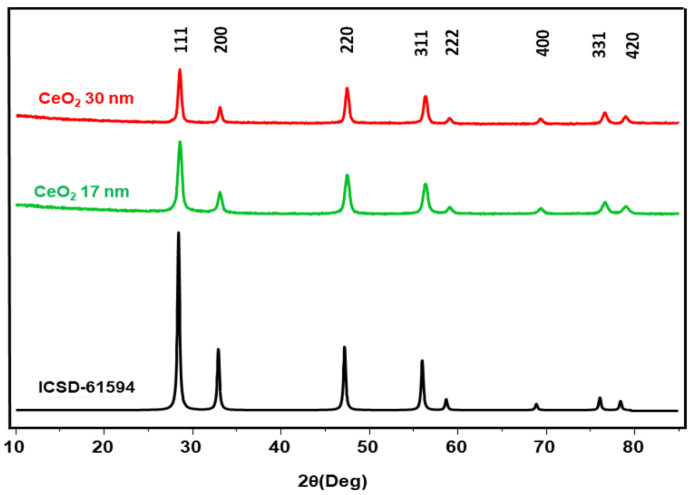
Powder X-ray diffractogram patterns for ceria nanoparticles (30 nm in red and 17 nm in green) and the referenced ceria face-centered cubic fluorite structure [42].

The powder X-ray diffraction (XRD) patterns of the as-synthesized 17 and 30 nm ceria NPs (Figure 8) show the existence of diffraction peaks at (111), (200), (220), (311), (222), (400), (331), and (420), which are well indexed with the face-centered cubic fluorite structure of ceria given in black. Crystallite sizes of 20 and 15 nm were determined from the XRD patterns of 30 and 17 nm nanoparticles by using the Scherrer formula [41]. Together, these features confirm the formation of a fluorite-structured ceria network.

**Figure 9:**
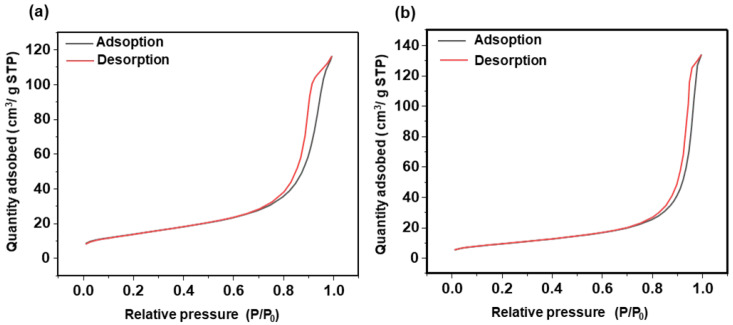
Nitrogen Adsorption and Desorption isotherms of the following: (**a**) 17 nm CeO_2_ nanoparticles; and (**b**) 30 nm CeO_2_ nanoparticles. The adsorbed nitrogen volume is given in cm^3^/g under standard temperature and pressure (STP) conditions.

The nitrogen adsorption–desorption isotherms recorded at 77 K for the CeO_2_ nanoparticles exhibit a reversible Type II isotherm, characteristic of gas adsorption on nonporous or macroporous surfaces (Figure 9) [37]. Additionally, both isotherms show a clear H3-type hysteresis loop. This H3 loop is generally associated with macroporous networks that are not completely filled during pore condensation, resulting in a cavitation-induced desorption branch at relative pressures (p/p_0_) close to saturation [37]. This confirms the presence of macroporous structures and interparticle voids within these nanoparticles. The BET surface areas were determined to be 49 m^2^/g for the 17 nm nanoparticles and 33 m^2^/g for the 30 nm nanoparticles.

**Figure 10:**
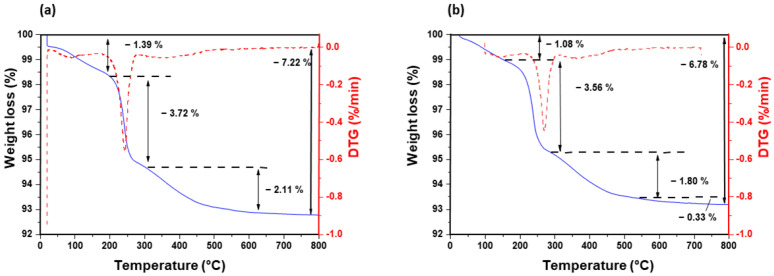
TGA (blue) and DTG curves (red) for CeO_2_ nanoparticles of 17 nm (**a**) and 30 nm (**b**).

TGA and DTG analyses were performed on CeO_2_ nanocrystals with mean sizes of 17 nm (Figure 10a) and 30 nm (Figure 10b). Both particle populations essentially exhibit the same thermal profile. A small, sharp weight decrease below ~150 °C is observed in both traces and is paired with a DTG maximum characteristic of desorption of physisorbed water very weakly bound surface to hydroxyls. The 17 nm sample shows a marginally larger loss here, reflecting its higher specific surface area. The principal DTG minimum, centered between ~220 °C and 260 °C, corresponds to the elimination of nitrate anions remnants (nitrates inherited from chemical synthesis) together with more strongly bound hydroxyl groups [43,44]. The peak temperature and shape of this event are virtually indistinguishable for the two materials, indicating identical surface chemistry despite the size difference. Above ~350 °C the mass declines gradually and no further DTG maxima appear until ~600 °C, where a broad, shallow minimum may correspond to the slow removal of chemisorbed nitrates and a minor Ce^4+^ → Ce^3+^ reduction accompanied by oxygen release [45]. This high-temperature process progresses smoothly to 800 °C without abrupt steps, confirming the robust fluorite lattice of both nanocrystalline powders [46]. XPS analyses performed on 17 nm and 30 nm ceria nanoparticles indeed confirm the presence of both Ce^3+^ and Ce^4+^ species, with a Ce^3+^/Ce^4+^ ratio of approximately 0.4 (Table S1). The characteristic Ce^4+^ shake-up satellite peak at 917.4 eV is also clearly observed.

### 3.2. Surface Roughness Evolution During Nanoparticle-Assisted Hyperpolishing of Hexagonal Small-Scale Zerodur Mirrors

#### 3.2.1. Surface Roughness Evolution via SiO_2_ Nanoparticle-Assisted Hyperpolishing

**Figure 11:**
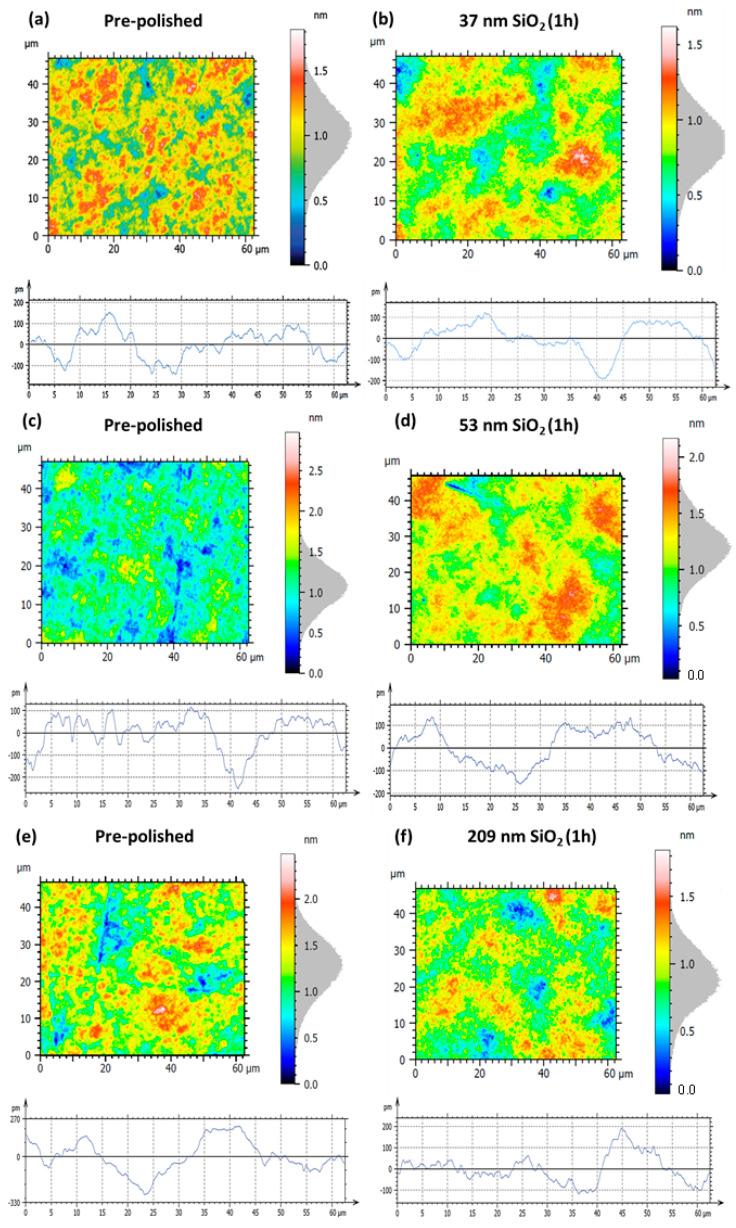
Surface and profile roughness measurements of the hexagonal Zerodur^®^ mirror: left panels (**a**,**c**,**e**) correspond to the initial pre-polished surface, while right panels (**b**,**d**,**f**) show the same surface after 1 h of hyperpolishing using SiO_2_ nanoparticles of 37 nm (**b**), 53 nm (**d**), and 209 nm (**f**) diameters, respectively.

To evaluate the effect of nanoparticle size on the polishing performance of Zerodur^®^ optical surfaces, slurries composed of as-synthesized SiO_2_ nanoparticles with diameters of 37, 53, and 209 nm were used in controlled hyperpolishing experiments. Interferometry was employed to monitor the surface evolution in terms of both area topography and profile roughness. The surface roughness parameters considered include the following: Sa (arithmetical mean height), which represents the average absolute deviation of the surface from the mean plane; Sq (root mean square height), corresponding to the standard deviation of surface heights; Pa (arithmetical mean peak height), defining the average height of surface peaks above the mean line; and Pq (root mean square peak height), measuring the root mean square of peak heights. In all analyses, the left panels depict the pre-polished baseline surfaces, while the right panels present the surfaces after 1 h of nanoparticle-assisted hyperpolishing. For each condition, the area roughness (Sa) is shown in the upper part of the panels, and the profile roughness (Pa) is displayed in the lower part of the panels. For the slurry containing 37 nm SiO_2_, the initial pre-polished surface exhibited an area roughness of Sa = 0.18 nm and Sq = 0.22 nm, with profile roughness values of Pq = 66.9 pm and Pa = 55.5 pm, which are consistent with fabrication-induced micro-roughness observed for other pre-polished Zerodur^®^ samples. After 1 h of hyperpolishing, Sa and Sq decreased to 0.15 nm (−16.7%) and 0.18 nm (−18.2%), respectively, indicating effective reduction in large-scale topographic features but with a slight increase in Pa (56.1, +1.10%) and Pq (71.2, +6.40%) (Table 2, Figure 11a,b). In the case of the 53 nm SiO_2_ slurry, the pre-polished surface displayed roughness values of Sa = 0.20 nm and Sq = 0.25 nm, with Pa = 62.5 pm and Pq = 79.0 pm. After 1 h of hyperpolishing (Figure 11d), the area roughness decreased to Sa = 0.17 nm (−15.0%) and Sq = 0.22 nm (−12.0%), reflecting slight improvement in overall flatness. However, the profile roughness exhibited divergent behavior: Pa slightly decreased to 77.8 pm (−1.50%) while Pq increased to 70.8 pm (+13.3%). For comparison, polishing with 209 nm SiO_2_ nanoparticles (Figure 11f) resulted in a more pronounced reduction in both area and profile roughness within just 1 h. The surface roughness improved from Sa = 0.23 nm, Sq = 0.29 nm, Pa = 92.1 pm, and Pq = 115.9 pm in the pre-polished state to Sa = 0.16 nm (−30.4%), Sq = 0.20 nm (−31.0%), Pa = 52.0 pm (−43.5%), and Pq = 66.9 pm (−42.3%) after hyperpolishing treatment.

**Figure 12:**
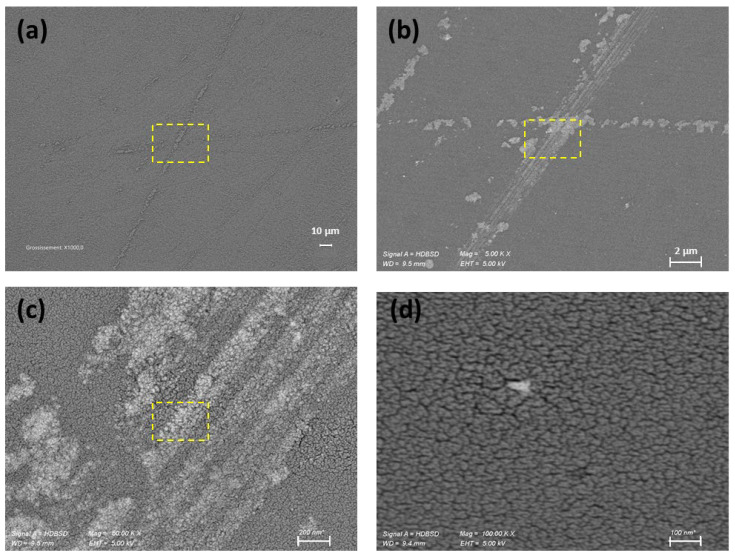
HRSEM images of the initial pre-polished Zerodur^®^ mirror surface with different magnifications showing prominent micro-scratches focused on the selected scratch region (in yellow) and revealing the microstructural texture and damage morphology or residual polishing grain. Scale bars = 10 μm (**a**), 2 μm (**b**), 200 nm (**c**), and 100 nm (**d**). Each subsequent image is a magnified view of the previous one, with the magnified area indicated by a yellow dashed rectangle.

**Figure 13:**
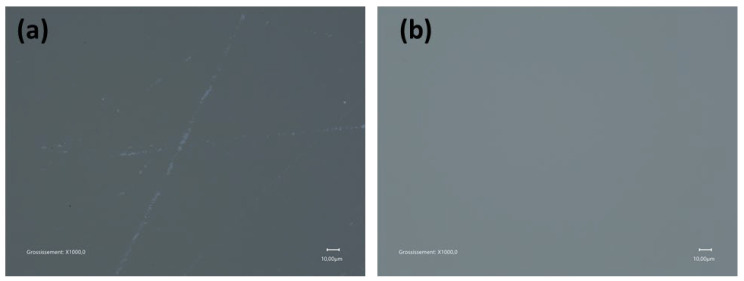
HRSEM images of the Zerodur^®^ mirror surface at 1000× magnification: (**a**) initial surface showing prominent micro-scratches prior to polishing; and (**b**) same area after 1 h of polishing with 209 nm SiO_2_. Scale bars = 10 μm.

In depth investigation of the initial surface via the topographic mapping (Figure 11a,c,e) and HRSEM images (Figure 12a–d) reveals the presence of fine filament-like features distributed across the mirror surface, which are indicative of residual polishing marks or micro-scratches originating from the initial fabrication steps. They are typically associated with mechanical abrasion during coarse polishing. Such features contribute significantly to the initial surface roughness and represent a critical barrier to achieving optical-grade smoothness. Following nanoparticle-assisted polishing, HRSEM observations (Figure 12) confirm the effective removal of these filamentary features. All nanoparticle sizes studied, 209, 53, and 37 nm SiO_2_, were successful in eliminating these residual scratches as shown in Figure 13b for 209 NPs and Figure S1a,b (Electronic Supporting Information (ESI)) for 53 and 37 nm SiO_2_ nanoparticles, respectively.

**Figure 14:**
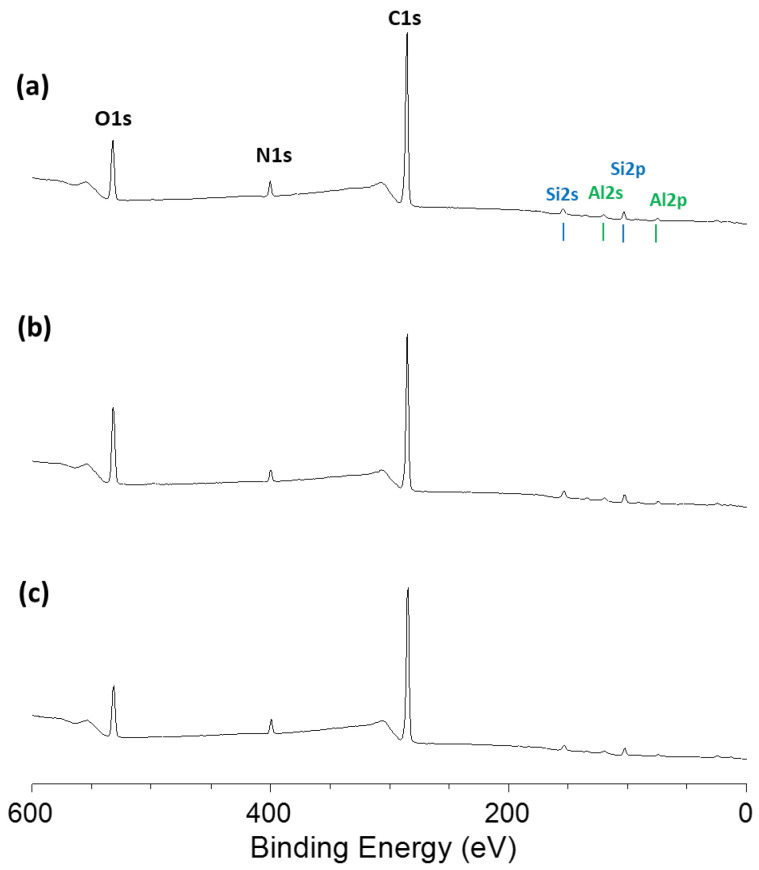
XPS surveyspectra of Zerodur^®^ surface after 1 h hyperpolishing with slurries containing SiO_2_ nanoparticle of the following: (**a**) 37 nm, (**b**) 53 nm, and (**c**) 209 nm.

Figure 14 presents the XPS survey spectra of Zerodur^®^ surfaces after the hyperpolishing with SiO_2_ nanoparticles of the three investigated sizes. Polishing with these nanoparticles results in a moderate decrease in carbon content, ranging from 4.5% to 12% depending on the particle size. This residual carbon originates from surface contamination and polishing residues, as Zerodur^®^ itself contains no intrinsic carbon. For the 53 nm nanoparticles, in addition to the most pronounced effect of reducing the carbon content (~12%), an increase in the aluminum signal of +69%, compared to the 18% increase observed with the other tested nanoparticles, was recorded. For any slurry for hyperpolishing, the resulting surface composition reveals the same chemical components with the appearance of Al, Si, C, N, and O peaks on the corresponding survey spectra, and small amounts of titanium and zirconium can be found in a close-up examination (Table S2, Electronic Supporting Information (ESI)). Whatever the nanoparticle’s size, the high-resolution analysis of the Al2p energetic region indicates that the oxidation state of Al is +3 as the observed binding energy of 74.9 eV is usually found in oxynitride glasses [47]. With the nanoparticles of 209 nm, the Si2p spectrum of the surface is the same as the one with the Zerodur^®^ substrate with a binding energy of 103.2 eV, which is associated with SiO_2_ chemical environment [48]. As the size of nanoparticles decreases, the Si2p peak shifts to low binding energy at 103.0 eV for nanoparticles of 53 nm and 102.7 eV for the ones of 30 nm.

These results demonstrate that all SiO_2_ nanoparticles tested are shown to be effective for improving the final state of the Zerodur^®^ substrate, reducing the filament structures that are still present after the pre-polishing procedure. Moreover, nanoparticle size influences the polishing performance, as already reported in the literature for other substrates [49,50,51,52,53], particularly with regard to the area (Sa, Sq) and profile roughness (Pa, Pq). After 1 h of polishing, all tested SiO_2_ slurries achieved reductions in area roughness. However, differences become apparent when looking at profile roughness parameters (Pa, Pq). While the 209 nm slurry led to a clear reduction in profile roughness indicating effective removal of micro-asperities, the 37 nm slurry resulted in a slight increase in Pq and Pa. For the 53 nm nanoparticles, the results appear intermediate: Pa increases, while Pq slightly decreases. Furthermore, XPS consistently showed across all three nanoparticle sizes that decreasing particle size leads to a systematic decrease in the Si2p peak binding energy, attesting to the formation of reduced SiOx (x < 2) phases, as previously reported by Alfonsetti et al. [54] This observation suggests a clear relationship between the size of the polishing silica nanoparticles and the reduction in silicic phases at the substrate surface; these phases likely originate from both the initial glass composition and possible degradation of the nanoparticles during the polishing process. However, the 53 nm nanoparticles show a final surface state slightly different from the two other nanoparticles’ sizes. Both XPS and interferometry analyses may suggest that if macro-scale smoothing occurred, localized micro-features remained or were unevenly modified for this size of nanoparticle [55]. Thus, the results highlight a clear improvement in surface flatness, with the Sa decreasing by 16.7% for the 37 nm slurry, 15.0% for the 53 nm slurry, and 30.4% for the 209 nm slurry, demonstrating the effectiveness of the hyperpolishing treatments and reflecting the greater ability of larger particles to eliminate surface asperities and defects introduced during earlier processing [56]. While the underlying tribological mechanisms were not the focus of this study, these observations remain consistent with the existing literature, which reports that larger nanoparticles tend to promote a stronger contribution of indentation-based mechanisms, whereas smaller nanoparticles favor area-based smoothing mechanisms [57]. This known size-dependent behavior provides a coherent explanation for the differences observed in the final surface quality. It also supports the idea that smaller particles, while effective for large-scale flattening, may require longer polishing times or optimized process conditions to fully address profile-level defects [58]. Indeed, extending the hyperpolishing by one more hour using 37 nm particles has resulted in an improvement in the profile metrics (Pa = 50.2 pm (−9.50%), Pq = 62.0 pm (−7.3%), with a slight reduction in Sa to 0.14 (−22.2%) while Sq is unchanged at 0.18 (−18.2%) (Figure S2 and Table S3, Electronic Supporting Information (ESI)), supporting the view that extended exposure enhances the chemo-mechanical smoothing action of the smaller nanoparticles and results in more uniform surface morphology.

#### 3.2.2. Surface Roughness Evolution via CeO_2_ Nanoparticle-Assisted Hyperpolishing

Based on previous studies that have demonstrated the efficiency of cerium oxide nanoparticles in CMP applications [20,59,60], the present section investigates their uses in the hyperpolishing of Zerodur^®^ mirrors. Specifically, two investigated nanoparticles, having the sizes of 17 nm and 30 nm, were evaluated to assess their polishing performance, leading to direct comparison of their effect with the above-described silica nanoparticle-based slurries (Section 3.2.1).

**Figure 15:**
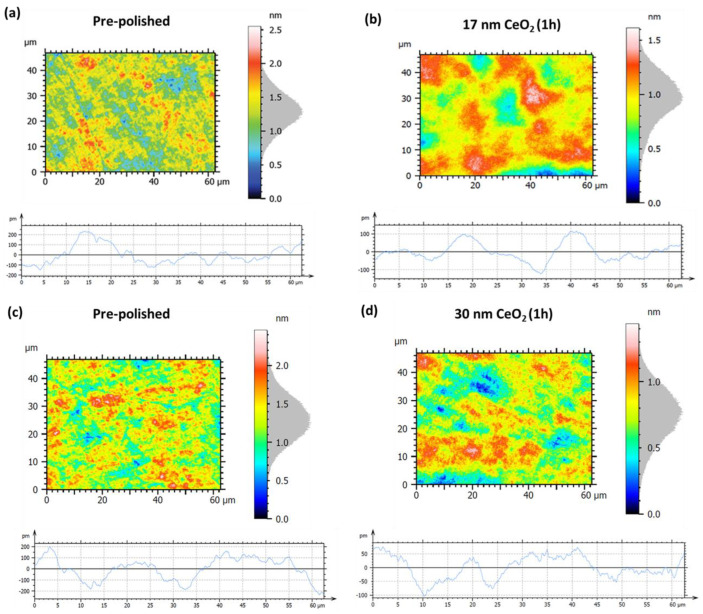
Surface and profile roughness measurements of the hexagonal Zerodur^®^ mirror for left panels: (**a**,**c**) are the initial pre-polished surfaces; and right panels: (**b**,**d**) are the same surfaces after 1 h of hyperpolishing using CeO_2_ nanoparticles of 17 nm (**b**) and 30 nm (**d**) diameter, respectively.

The initial pre-polished surface (Figure 15a) exhibits an area roughness of Sa = 0.21 nm and Sq = 0.26 nm (Table 3). Corresponding line profile measurements yield Pa = 71.8 pm and Pq = 90.3 pm, and reflect the presence of pronounced surface asperities and nanoscale irregularities. After one hour of hyperpolishing with the 17 nm CeO_2_ slurry (Figure 15b), a significant improvement in surface quality is achieved, with area roughness values reduced to Sa = 0.16 nm (−23.8%) and Sq = 0.21 nm (−19.2%). Profile roughness values show an even more significant reduction, with Pq falling to 51.1 pm (−43.4%) and Pa to 39.6 pm (−44.8%), which is consistent with enhanced suppression of peak-to-valley surface variations. Further enhancement is achieved using 30 nm CeO_2_ nanoparticles, as shown in Figure 15d. After 1 h of hyperpolishing, the area roughness is reduced to Sa = 0.15 nm (−34.8%) and Sq = 0.19 nm (−32.1%). Profile parameters also improve, with Pq = 41.9 pm (−58.4%) and Pa = 35.6 pm (−57.7%), representing superior attenuation of fine-scale surface features. Although the area roughness values are similar between the two ceria-polished samples, the profile metrics slightly favor the 30 nm slurry, suggesting more uniform micro-asperity removal and improved control over surface texture. This is consistent with prior findings by Cui et al. [24], who reported enhanced polishing performance with larger ceria nanoparticles under comparable conditions.

**Figure 16:**
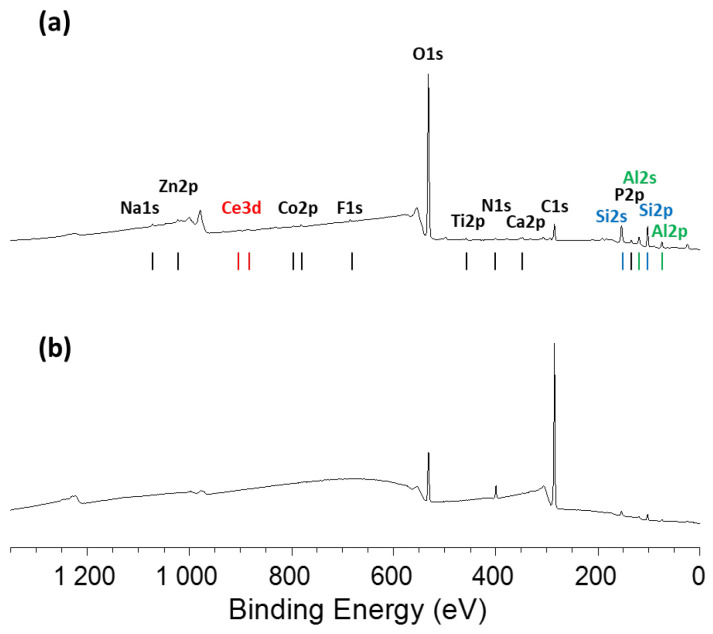
XPS survey spectrum of Zerodur^®^ surface after 1 h hyperpolishing with two slurries containing CeO_2_ nanoparticles of the following: (**a**) 17 nm and (**b**) 30 nm.

Examination of the XPS survey spectra after polishing with CeO_2_ nanoparticles reveals a greater presence of substrate-specific elements (such as Zn, Co, Ti, and Mg) compared to polishing with SiO_2_ nanoparticles (Figure 16).

Regardless of nanoparticle size, polishing leads to a reduction in the relative surface carbon content (Table S2, Electronic Supporting Information (ESI)). This effect is much more pronounced with the smaller nanoparticles. Indeed, using the slurry containing 17 nm CeO_2_ nanoparticles, surface carbon is almost entirely eliminated, with an atomic concentration reduced to approximately 9%, enhancing the detectability of the aluminum signal, which remains in the +3 oxidation state. Moreover, the silicon concentration on the surface increases by a factor of five when polishing with the 17 nm CeO_2_ slurry. The Si2p binding energy remains characteristic of the SiO_2_ phases, as expected from the composition of the Zerodur^®^ substrate. As for cerium, only trace (~0.2 at.%) were detected on the polished surfaces, regardless of the nanoparticle size. Further insight from the Ce3d core level spectrum reveals a predominant Ce^3+^ chemical environment (Figure S3, Electronic Supporting Information (ESI)), suggesting partial reduction in CeO_2_ nanoparticles during the polishing process. The shake-up features typically associated with Ce^4+^ in CeO_2_ are largely absent, although a broad shoulder at 917.4 eV confirms the presence of a small surface fraction of Ce^4+^. Specifically, in the case of the 17 nm nanoparticles, the quantified Ce^3+^/Ce^4+^ ratio reaches approximately 19 on the polished surface, which is attributed to the presence of a few residual ceria nanoparticles remaining after hyperpolishing. In comparison, the initial CeO_2_ nanoparticles exhibit a much lower Ce^3+^/Ce^4+^ ratio of 0.4 prior to their use. (Table S1, Electronic Supporting Information (ESI)) [61].

Note that similar surface roughness values (S_a_ ≈ 0.15 nm) after one hour of treatment were obtained with CeO_2_ and SiO_2_ nanoparticles of equivalent size (~30 nm) under identical CMP conditions even though the initial surface quality was not the same. However, a distinction emerges in surface quality when assessed using profile-based roughness metrics, reflecting variations in the effectiveness of the polishing process in reducing peak height and surface irregularities between SiO_2_ and CeO_2_ nanoparticles. Specifically, CeO_2_ nanoparticles yield significantly lower arithmetic roughness values, with Pa = 35.6 pm and Pq = 41.9 pm, compared to Pa = 56.1 pm and Pq = 71.2 pm for SiO_2_ abrasives. These results highlight the polishing capability of CeO_2_ at the nanoscale, likely attributable to its combined mechanical and chemical interaction with the Zerodur^®^, which is conducive to uniform material removal and smooth final surface morphology. This effect has also been reported in studies realized using ceria nanoparticles on other substrates [24,62].

#### 3.2.3. Surface Roughness Evolution via Synergic Effect of Nanoparticle

**Figure 17:**
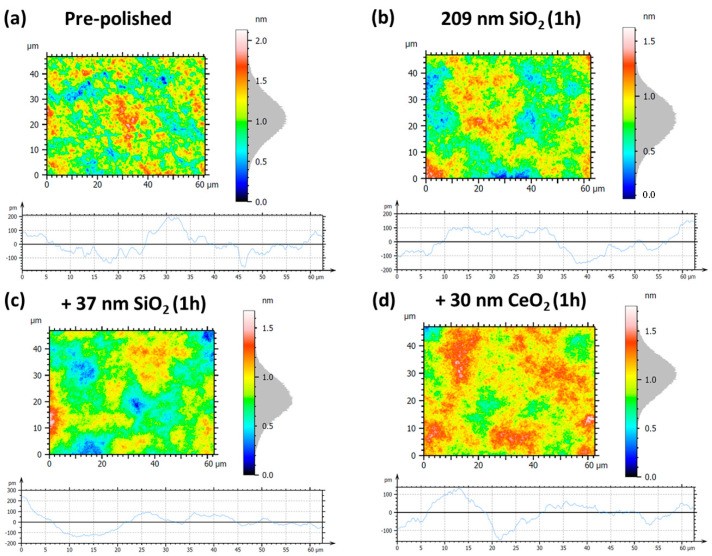
Hexagonal Zerodur^®^ mirror surface roughness measurement and profile roughness for the (**a**) initial pre-polished surface and with (**b**) 1 h hyperpolishing with 209 nm SiO_2_ nanoparticle slurry followed by (**c**) 1 h hyperpolishing with 37 nm SiO_2_ nanoparticle slurry followed by (**d**) 1 h hyperpolishing with 30 nm CeO_2_ nanoparticle slurry.

Based on the results obtained during mirror hyperpolishing using a single slurry of nanoparticles, we identified optimal outcomes associated with each abrasive. Notably, 209 nm SiO_2_ exhibited a rapidly reducing micro-scale roughness, while both 37 nm SiO_2_ and 30 nm CeO_2_ produced comparable Sa values, with CeO_2_ offering superior surface profile metrics. These findings motivated the implementation of a hyperpolishing sequence combining the successive employement of 209 nm, 37 nm SiO_2_, and 30 nm CeO_2_ nanoparticles to investigate potential synergistic effects (Figure 17, Table 4). The initial surface shows the following parameters: Sa = 0.20 nm, Sq = 0.25, Pa = 60.6, and Pq = 76.4 pm (Figure 17a).

Polishing with the slurry containing 209 nm SiO_2_ (Figure 17b) reduces Sa to 0.15 nm (−25.0%) and Sq = 0.20 nm (−20.0%), which is consistent with earlier observations on individual mirrors, but Pa and Pq increases to 68.7 (+13.4%) and 78.8 pm (+3.10%), respectively. The additional following treatment with 37 nm of SiO_2_ (Figure 17c) leads to similar Sa of 0.15 (−25.0%) nm and slight reduction in Sq to 0.19 nm (−24.0%) and a slight improvement in profile metrics with Pa = 57.4 pm (−5.30%) and Pq = 74.7 pm (−22.7%). The following additional surface treatment with the slurry containing 30 nm of CeO_2_ achieves the most significant reduction in Sa equal to 0.14 nm (−30.0%) and Sq = 0.18 nm (−28.0%) and Pa = 46.3 pm (−23.6%) and Pq of 59.0 pm (−22.7%) (Figure 17d), which is in agreement with individual separated trials described in Section 3.2.2.

**Figure 18:**
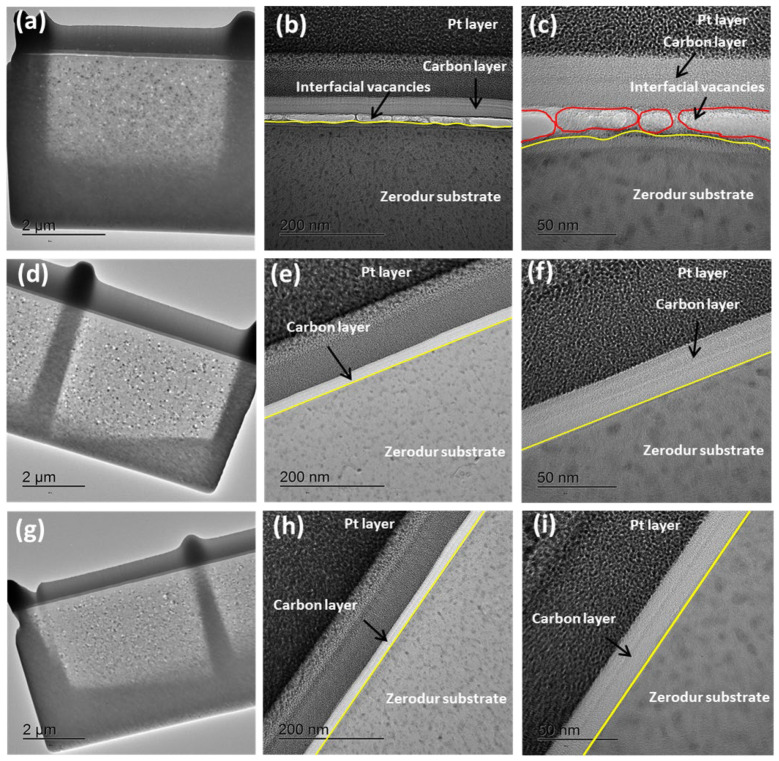
HR TEM cross-sectional images of Zerodur mirror surfaces: (**a**–**c**) pre-polished surface; (**d**–**f**) after 1 h of polishing with 37 nm SiO_2_ nanoparticles; (**g**–**i**) after synergistic polishing using a sequential combination of 209 nm SiO_2_, 37 nm SiO_2_, and 30 nm CeO_2_ nanoparticles. Insets highlight the protective Pt and carbon layers used during FIB preparation. The yellow lines indicate the interface between the Zerodur^®^ substrate and the deposited carbon film, while the red lines highlight interfacial vacancies.

Figure 18 presents HR TEM cross-sectional images of the Zerodur^®^ mirror surface: initial pre-polished (a–c), hyperpolished with 30 nm SiO_2_ nanoparticles (Figure 18d–f), and subjected to synergistic hyperpolishing using successive slurries of three nanoparticle types (Figure 18g–i). In Figure 18b,c, the interface between the protective carbon layer and the Zerodur substrate is visibly irregular, as highlighted by yellow lines. These surface fluctuations correspond to residual topographical defects from earlier mechanical processing. Additionally, clear signs of poor adhesion and interfacial vacancies (represented in red in Figure 18c) are observed between the carbon coating and the unpolished Zerodur^®^ surface, indicating non-uniform surface energy distribution likely caused by rough, damaged regions. In contrast, polishing with 37 nm SiO_2_ nanoparticles (Figure 18e,f) results in a noticeably smoother interface, with reduced surface irregularities and more uniform contact between the carbon layer and the substrate. Similar improvements are observed in the samples polished with the synergistic nanoparticle slurry (Figure 18h,i), achieved through a sequential combination of nanoparticles: 209 nm SiO_2_, 37 nm SiO_2_, and 30 nm CeO_2_. These images demonstrate a continuous, well-adhered carbon layer and a highly uniform Zerodur^®^ surface, indicating enhanced surface planarity and, therefore, less roughness. The absence of interfacial detachment and morphological distortion in the polished samples demonstrates the effectiveness of hyperpolishing with nanoparticles of either SiO_2_ or CeO_2_ in removing nanoscale defects and enhancing coating compatibility.

### 3.3. Surface Roughness Evolution During Nanoparticle-Assisted Hyperpolishing of Spherical Large-Scale Zerodur Mirrors

**Figure 19:**
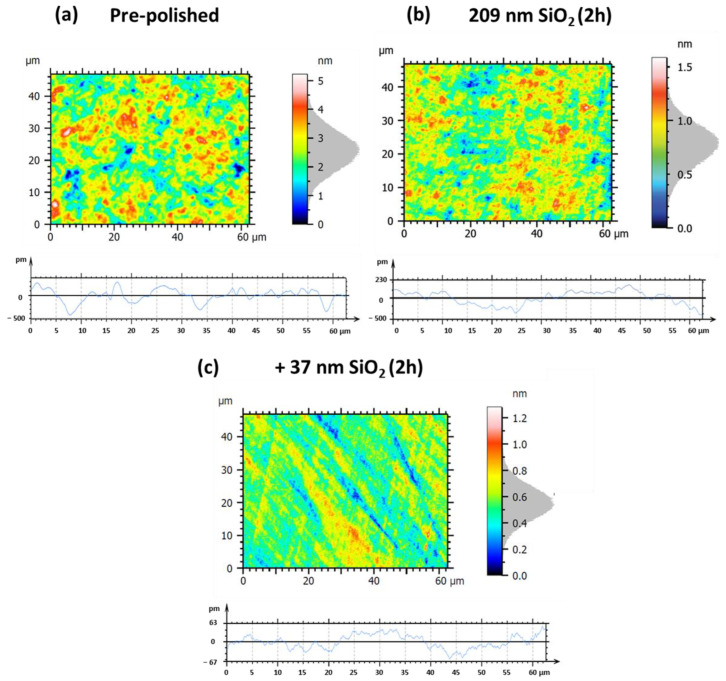
Large spherical profile Zerodur^®^ mirror surface roughness measurement and profile roughness for the following: (**a**) initial pre-polished surface, (**b**) after 2 h hyperpolishing with 209 nm SiO_2_ nanoparticle slurry, followed by (**c**) 2 h hyperpolishing with 37 nm SiO_2_ nanoparticle slurry.

**Figure 20:**
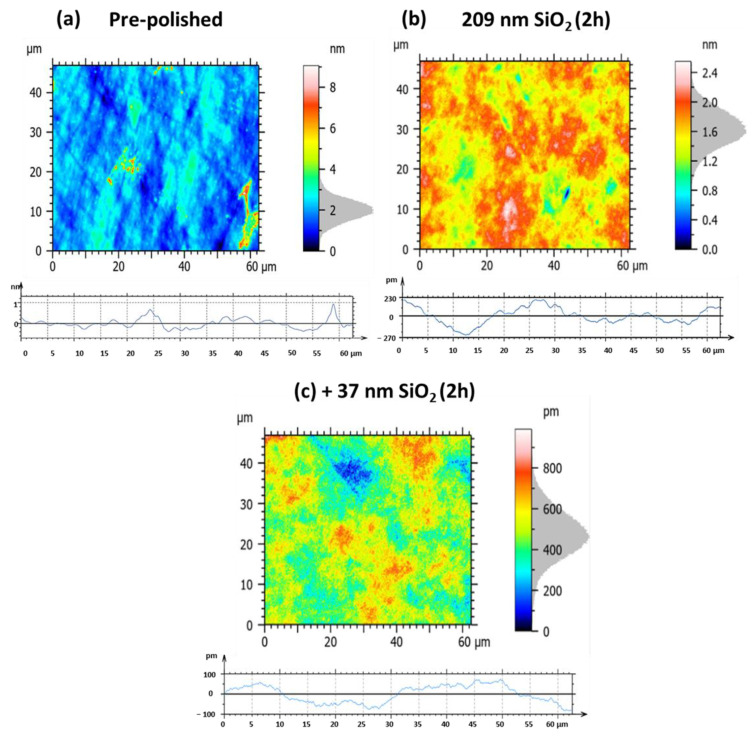
Large plano Zerodur^®^ mirror surface roughness and profile roughness for the following: (**a**) initial pre-polished surface, (**b**) after 2 h hyperpolishing with 209 nm SiO_2_ nanoparticle slurry, followed by (**c**) 2 h hyperpolishing with 37 nm SiO_2_ nanoparticle slurry.

The various experiments conducted on small hexagonal Zerodur^®^ mirrors with a circumscribed diameter of 30 mm have demonstrated that both silica and ceria nanoparticles can significantly improve the surface quality of this substrate. Furthermore, among the different silica nanoparticles tested, we identified notable differences in behavior between the 209 and 37 nm SiO_2_ nanoparticles: the larger ones were more effective in rapidly reducing surface defects, while the smaller ones enabled achieving superior surface quality, albeit over longer hyperpolishing times. In addition, we demonstrated the benefit of adopting a synergistic approach by alternately combining these different silica particle sizes during the polishing process. As hyperpolishing must be scaled to accommodate larger mirrors in response to the optical requirements of exoplanet imaging, we extended this study to mirrors with increased diameters of 135 mm to evaluate process efficiency. To this end, and in order to limit the number and duration of experiments, we chose to focus exclusively on silica nanoparticles, although ceria nanoparticles also prove to be excellent candidates for polishing Zerodur^®^. Building on previously obtained results, we decided to first use the 209 nm silica nanoparticles, followed by the 37 nm particles. In both cases, the polishing time was extended to two hours. These conditions were applied for the ultra-polishing of Zerodur^®^ mirrors with either a spherical profile or a plano surface. Figure 19 and Table 5 relate to the hyperpolishing of a spherical profile Zerodur^®^ mirror, while Figure 20 and Table 6 present results for a plano Zerodur^®^ mirror. The roughness data summarized in Table 5 and Table 6 clearly illustrate the progressive improvement in surface quality achieved through sequential polishing stages on Zerodur^®^ mirror substrates with different profiles.

Initially, the pre-polished samples exhibit considerable surface roughness and micro-scale irregularities. For the spherical mirror (Table 5a, Figure 19a), the roughness parameters are Sa = 0.53 nm, Sq = 0.67 nm, Pa = 0.11 nm, and Pq = 0.14 nm. For the plano mirror (Table 6a, Figure 20a), the initial roughness is Sa = 0.46 nm, Sq = 0.67 nm, Pa = 170 pm, and Pq = 220 pm, clearly indicating surface conditions unsuitable for precision optical applications. Following a 2 h hyperpolishing step using a slurry containing 209 nm SiO_2_ nanoparticles (samples b), both mirrors show significant reductions in roughness. The spherical mirror’s values of Sa and Sq decrease to 0.15 nm (−71.7%) and 0.19 nm (−71.6%), respectively (Table 5b, Figure 19b), while the plano mirror shows Sa and Sq values of 0.19 nm (−58.7%) and 0.24 nm (−64.2%) (Table 6b, Figure 20b). However, in both cases, the profile roughness parameters Pa (74.0 pm (−32.7%) to 83.2 pm (−51.1%)) and Pq (87.0 pm (−37.9%) to 103 pm (−53.2%)) remain relatively elevated, indicating persistent micro-scale surface irregularities. An additional 2 h hyperpolishing step with smaller 37 nm SiO_2_ nanoparticles (samples c) markedly improves surface finish for both mirror types. The spherical mirror reaches Sa = 0.10 nm (−81.1%), Sq = 0.13 nm (−80.6%), Pa = 19.3 pm (−82.5%), and Pq = 23.0 pm (−83.6%) (Table 5c, Figure 19c), while the plano mirror achieves Sa = 90.1 pm (−80.4%), Sq = 114.5 pm (−82.9%), Pa = 39.0 pm (−77.1%), and Pq = 42.8 pm (−80.5%) (Table 6c, Figure 20c). These results underscore the critical influence of particle size on polishing efficiency, demonstrating that smaller SiO_2_ nanoparticles substantially enhance the ability to achieve ultra-smooth surfaces. Consequently, this optimized, synergistic polishing protocol reliably delivers sub-angstrom roughness levels essential for advanced optical applications requiring extreme precision.

## 4. Conclusions

In summary, this study focused on the hyperpolishing of Zerodur^®^ mirror surfaces by using different slurries containing SiO_2_ and CeO_2_ nanoparticles of various sizes, along with the successive employment and evaluation of their size and composition on the treatment efficiency. To enable meaningful comparisons, we initially selected a set of hexagonal mirrors with a circumscribed diameter of 30 mm. Each mirror underwent one hour of hyperpolishing with slurries containing only the specific nano-abrasive under investigation:

*(i)*  This first experimental phase allowed us to demonstrate that all tested nanoparticle sizes led to a significant improvement in surface quality. More specifically, both the largest (209 nm) and smallest (37 nm) SiO_2_ nanoparticles produced very similar final surface states, with a performance advantage for the larger particles. However, this advantage could easily be compensated for by extending the polishing time for the smaller nanoparticles; achieving better results after two hours of polishing instead of one.
*(ii)*  The slurry containing CeO_2_ nanoparticles of 30 nm showed a noticeably better surface quality and higher hyperpolishing efficiency in comparison to the results obtained with the 37 nm SiO_2_ particles.
*(iii)*  We then evaluated a sequential polishing approach using different SiO_2_ nanoparticle sizes (209 nm followed by 37 nm), and then CeO_2_ nanoparticles (30 nm). This sequential strategy further confirmed the trends observed in the single-slurry tests.
*(iv)*  We focused next on validating one of the most promising polishing protocols on larger optics. Based on the results obtained with the small 30 mm mirrors, where sequential polishing with SiO_2_ nanoparticles (209 nm followed by 37 nm) proved effective, we scaled up the process to larger Zerodur^®^ mirrors (135 mm in diameter) with both spherical and plano surface profiles. These geometries are representative of optics intended for high-precision applications such as exoplanet detection and imaging. For this scale-up, we retained the sequential SiO_2_-based polishing protocol, applying two hours of polishing with 209 nm particles followed by two additional hours with 37 nm particles. This process yielded optics with excellent surface quality, achieving area roughness (Sa) of 1 Å for the spherical mirrors and 0.9 Å for the plano mirrors, a remarkable result considering the total hyperpolishing time was limited to just 4 h.

These findings clearly demonstrate the potential of nanoparticle-assisted CMP processes, where the use of well-controlled nanoparticle sizes enables the achievement of ultra-smooth surface finishes, pushing the limits of surface quality to sub-nanometric levels. They also highlight a promising pathway for extending such hyperpolishing techniques to other substrates of interest for demanding scientific and industrial applications.

## Figures and Tables

**Table 1:** Experimental Parameters for the controlled synthesis of SiO_2_ nanoparticles of 37, 53, and 209 nm.

Size	V_EtOH_	V_H2O_	V_TEOS_	Temperature
209 ± 11 nm	400 mL	8 mL	12 mL	20 °C
53 ± 6 nm	200 mL	200 mL	12 mL	30 °C
37 ± 5 nm	400 mL	8 mL	12 mL	75 °C

**Table 2:** Specific roughness parameters for hexagonal Zerodur^®^ mirror before and after hyperpolishing with 37 nm, 53 nm, and 209 nm SiO_2_-based slurries.

Figure 11 Panels	Sa	Sq	Pa	Pq
(a) pre-polished	0.18 nm	0.22 nm	55.5 pm	66.9 pm
(b) 37 nm SiO_2_, 1 h	0.15 nm (−16.7%)	0.18 nm (−18.2%)	56.1 pm (+1.10%)	71.2 pm (+6.40%)
(c) pre-polished	0.20 nm	0.25 nm	79.0 pm	62.5 pm
(d) 53 nm SiO_2_, 1 h	0.17 nm (−15.0%)	0.22 nm (−12.0%)	77.8 pm (−1.50%)	70.8 pm (+13.3%)
(e) pre-polished	0.23 nm	0.29 nm	92.1 pm	115.9 pm
(f) 209 nm SiO_2_, 1 h	0.16 nm (−30.4%)	0.20 nm (−31.0%)	52.0 pm (−43.5%)	66.9 pm (−42.3%)

**Table 3:** Specific roughness parameters for Zerodur mirror before and after hyperpolishing with 17 and 30 nm CeO_2_-based slurries.

Figure 15 Panels	Sa	Sq	Pa	Pq
(a) pre-polished	0.21 nm	0.26 nm	71.8 pm	90.3 pm
(b) 17 nm CeO_2_, 1 h	0.16 nm (−23.8%)	0.21 nm (−19.2%)	39.6 pm (−44.8%)	51.1 pm (−43.4%)
(c) pre-polished	0.23 nm	0.28 nm	84.2 pm	100.8 pm
(d) 30 nm CeO_2_, 1 h	0.15 nm (−34.8%)	0.19 nm (−32.1%)	35.6 pm (−57.7%)	41.9 pm (−58.4%)

**Table 4:** Specific roughness parameters for Zerodur^®^ mirror hyperpolished successively with 209 nm SiO_2_, then 30 nm SiO_2_, and then 30 nm CeO_2_-based slurry.

Figure 17 Panels	Sa	Sq	Pa	Pq
(a) pre-polished	0.20 nm	0.25 nm	60.6 pm	76.4 pm
(b) 209 nm SiO_2_	0.15 nm (−25.0%)	0.20 nm (−20.0%)	68.7 pm (+13.4%)	78.8 pm (+3.10%)
(c) +37 nm SiO_2_	0.15 nm (−25.0%)	0.19 nm (−24.0%)	57.4 pm (−5.30%)	74.7 pm (−2.20%)
(d) +30 nm CeO_2_	0.14 nm (−30.0%)	0.18 nm (−28.0%)	46.3 pm (−23.6%)	59.0 pm (−22.7%)

**Table 5:** Specific roughness parameters for large spherical profile Zerodur^®^ mirror hyperpolished successively by 209 nm SiO_2_ and then by 37 nm SiO_2_-based slurries.

Figure 19 Panels	Sa	Sq	Pa	Pq
(a) pre-polished	0.53 nm	0.67 nm	0.11 nm	0.14 nm
(b) 209 nm SiO_2_, 2 h	0.15 nm (−71.7%)	0.19 nm (−71.6%)	74.0 pm (−32.7%)	87.0 pm (−37.9%)
(c) +37 nm SiO_2_, 2 h	0.10 nm (−81.1%)	0.13 nm (−80.6%)	19.3 pm (−82.5%)	23.0 pm (−83.6%)

**Table 6:** Specific roughness parameters for large plano Zerodur^®^ mirror successively hyperpolished by 209 nm SiO_2_ and then 37 nm SiO_2_-based slurries.

Figure 20 Panels	Sa	Sq	Pa	Pq
(a) pre-polished	0.46 nm	0.67 nm	0.17 nm	0.22 nm
(b) 209 nm SiO_2_, 2 h	0.19 nm (−58.7%)	0.24 nm (−64.2%)	83.2 pm (−51.1%)	103 pm (−53.2%)
(c) +37 nm SiO_2_, 2 h	90.1 pm (−80.4%)	114.5 pm (−82.9%)	39.0 pm (−77.1%)	42.8 pm (−80.5%)

## Data Availability

The original contributions presented in this study are included in the article/Supplementary Materials. Further inquiries can be directed to the corresponding author(s).

## Supplementary Materials

The following supporting information can be downloaded at: https://www.mdpi.com/article/10.3390/nano15181391/s1, Figure S1. HRSEM images of the Zerodur^®^ mirror surface at 1000× magnification: (**a**) surface after 1 h of polishing with 53 nm SiO_2_. Scale (**b**) surface after 1 h of polishing with 37 nm SiO_2_. Scale bars = 10 µm.; Figure S2. Hexagonal Zerodur^®^ mirror surface roughness measurement and profile roughness for (**a**), initial pre-polished surface and after 2 h hyperpolishing with (**b**) 37 nm SiO_2_ NPs.; Figure S3. XPS Ce3d spectrum Zerodur^®^ mirror surface after 1 h hyperpolishing with 17 nm CeO_2_ NPs slurry.; Table S1. Ce^III^/Ce^IV^ ratio for 17 nm and 30 nm CeO_2_ nanoparticles’ surface and Zerodur^®^ surface after polishing.; Table S2. XPS composition table of elements for Zerodur ^®^ pre-polished substrate and after hyperpolishing with nanoparticles of different sizes and compositions.; Table S3. Specific roughness parameters for hexagonal Zerodur^®^ mirror before and after 2 h hyperpolishing with 37 nm SiO_2_-based slurries.

## Abbreviations

The following abbreviations are used in this manuscript:

ATR	Attenuated Total Reflectance
BET	Brunauer–Emmett–Teller
CMP	Chemical Mechanical Polishing
DLS	Dynamic Light Scattering
DTG	Derivative Thermogravimetry
FTIR	Fourier Transform Infrared Spectroscopy
HRSEM	High-Resolution Scanning Electron Microscopy
HRTEM	High-Resolution Transmission Electron Microscopy
IR	Infrared Spectroscopy
MAS	Magic Angle Spinning
NASA	National Aeronautics and Space Administration
NMR	Nuclear Magnetic Resonance
NPs	Nanoparticles
Pa	Arithmetic Mean Height
Pq	Root Mean Square Height
Sa	Areal Arithmetic Mean Height
SEM	Scanning Electron Microscopy
Sq	Root Mean Square Roughness
TEM	Transmission Electron Microscopy
TEOS	Tetraethyl Orthosilicate
TGA	Thermogravimetric Analysis
XPS	X-ray Photoelectron Spectroscopy
XRD	X-ray Diffraction
