# Supplementary material for: Chemical Mechanical Polishing of Zerodur® Using Silica and Ceria Nanoparticles: Toward Ultra-Smooth Optical Surfaces
Source: Nanomaterials (Basel). 2025 Sep 10;15(18):1391. doi: 10.3390/nano15181391 (PMC12472406; doi:10.3390/nano15181391)
Supplement: Supplementary file 1 [file nanomaterials-15-01391-s001.zip › nanomaterials-3817491-supplementary.pdf]

Electronic Supplementary Information

Chemical Mechanical Polishing of Zerodur® Using Silica and Ceria Nanoparticles: Toward Ultra-Smooth Optical Surfaces

Houda Bellahsene [1], Saad Sene [1], Gautier Félix [1], Nicolas Fabregue [1], Michel Marcos [2], Arnaud Uhart [3], Jean-Charles Dupin [3], Erwan Oliviero [1], Joulia Larionova [1], Marc Ferrari [2,*] and Yannick Guari [1,*]

[1] Institut Charles Gerhardt Montpellier (ICGM), Centre National de la Recherche Scientifique (CNRS), École Nationale Supérieure de Chimie de Montpellier (ENSCM), University of Montpellier (UM), 34293 Montpellier, France; houda.bellahsene@etu.umontpellier.fr (H.B.); saad.sene@umontpellier.fr (S.S.); gautier.felix@umontpellier.fr (G.F.); nicolas.fabregue@umontpellier.fr (N.F.); erwan.oliviero@umontpellier.fr (E.O.); joulia.larionova@umontpellier.fr (J.L.)

[2] Laboratoire d'Astrophysique de Marseille (LAM), Centre National de la Recherche Scientifique (CNRS), Centre National d'Etudes Spatiales (CNES) Aix Marseille University (AMU), 13013 Marseille, France; michel.marcos@lam.fr

[3] Institut des Sciences Analytiques et de Physico-Chimie pour l'Environnement et les Matériaux (IPREM), Centre National de la Recherche Scientifique (CNRS), Institut Mines-Télécom Mines Alès (IMT), Université de Pau et des Pays de l'Adour (UPPA), 64053 Pau, France; arnaud.uhart@univ-pau.fr (A.U.); jean-charles.dupin@univ-pau.fr (J.-C.D.)

* Correspondence: marc.ferrari@osupytheas.fr (M.F.); yannick.guari@umontpellier.fr (Y.G.); Tel.: +33-491-055-900 (M.F.); +33-448-792-085 (Y.G.)

Tables

Table S1. Ce^{III}/Ce^{IV} ratio for 17nm and 30nm CeO_2 nanoparticles surface and Zerodur® surface after polishing.

	17nm nanoparticles	Zerodur® after hyperpolishing with 17 nm CeO_2 nanoparticles	30nm nanoparticles	Zerodur® after hyperpolishing with 30 nm CeO_2 nanoparticles
Ce^{III}/Ce^{IV}	0.4	19	0.38	-

Table S2. XPS composition table of elements for Zerodur ® pre-polished substrate and after hyperpolishing with nanoparticles of different sizes and compositions.

		XPS atomic percentage of elements															
	Size	%C	%O	%Al	%N	%Na	%P	%Si	%Ce	%Ti	%Zr	%Ca	%Co	%Mg	%F	%K	%Zn
Zerodur ®*	-	78.4	11.3	1.6	4.1	0.1	0.5	4.0	-	-	-	-	-	-	-	-	-
SiO_2	37nm	74.9	13.9	1.9	3.9	0.1	0.5	4.6	-	0.1	0.1	-	-	-	-	-	-
	53nm	68.9	17.3	2.7	3.7	0.2	1.0	6.0	-	0.1	0.1	-	-	-	-	-	-

	209nm	69.7	18.6	1.9	3.7	0.1	0.6	5.2	-	0.1	0.1	-	-	-	-	-	-
CeO_2	17nm	5.9	56.8	9.3	0.3	0.6	2.5	21.2	0.2	0.3	0.2	0.7	0.2	0.5	0.6	0.6	0.1
	30nm	74.3	14.1	2.0	4.0	0.1	0.5	4.3	0.2	0.1	0.1	0.1	0.1	0.1	0.1	-	-

Table S3. Specific roughness parameters for hexagonal Zerodur® mirror before and after 2h hyperpolishing with 37 nm SiO_2 based slurries.

Sample	Sa	Sq	Pa	Pq
a	0.18 nm	0.22 nm	55.5 pm	66.9 pm
b	0.14 nm	0.18 nm	50.2 pm	62.0 pm

Figures

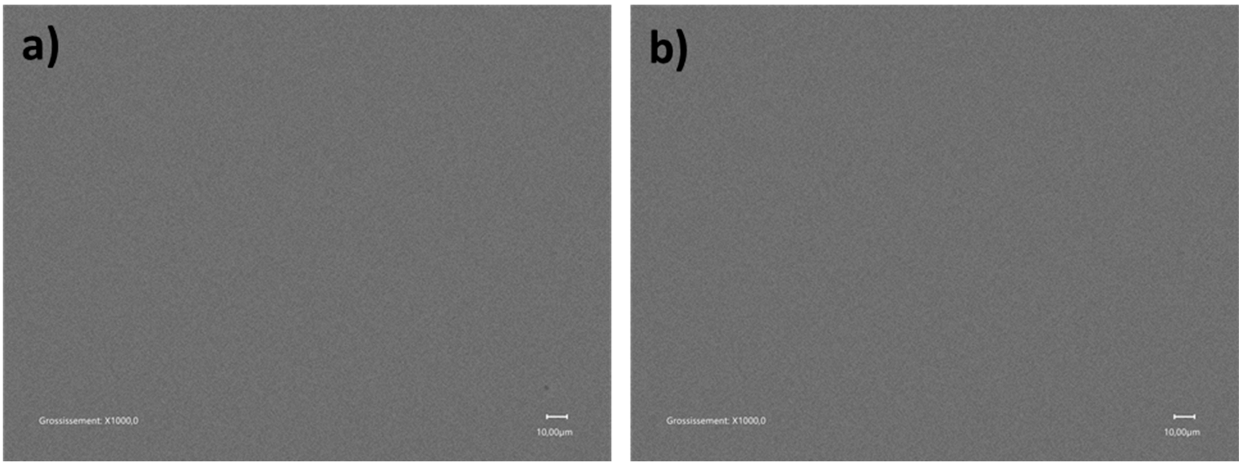

Figure S1. HRSEM images of the Zerodur® mirror surface at 1000× magnification: (a) surface after 1 hour of polishing with 53 nm SiO_2. Scale b) surface after 1 hour of polishing with 37 nm SiO_2. Scale bars = 10 µm.

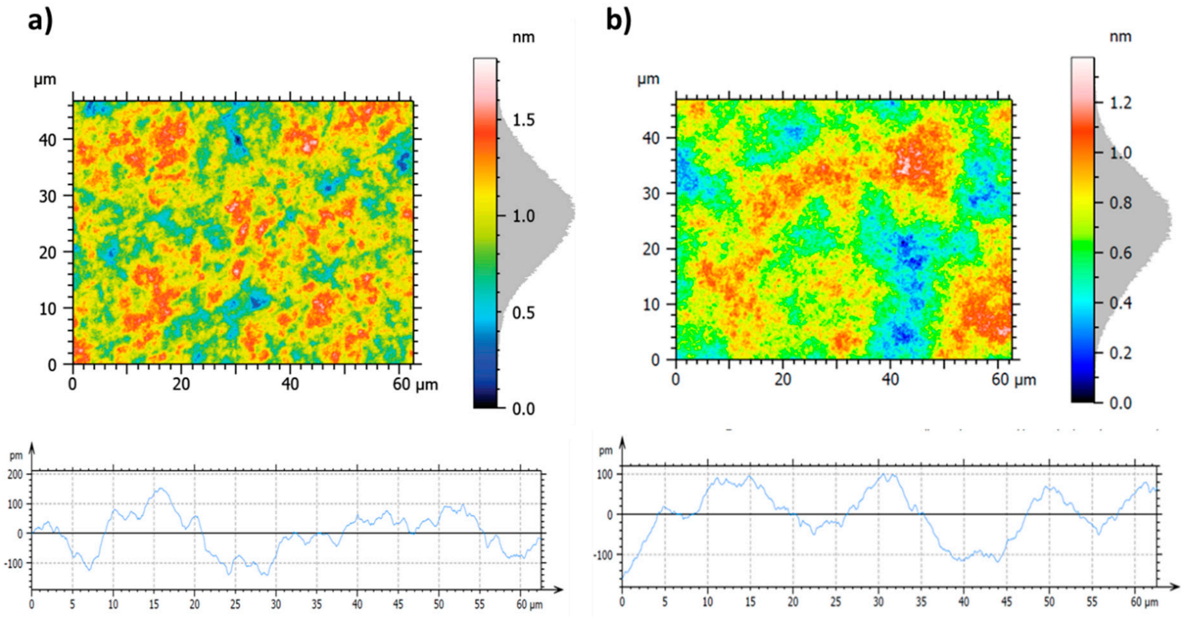

Figure S2. Hexagonal Zerodur® mirror surface roughness measurement and profile roughness for a), initial pre-polished surface and after 2h hyperpolishing with b) 37 nm SiO_2 NPs.

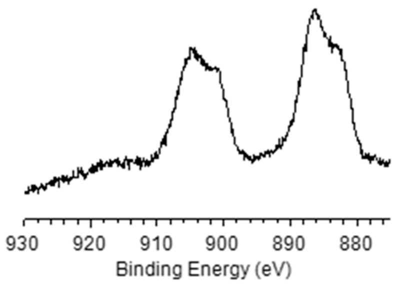

Figure S3. XPS Ce3d spectrum Zerodur® mirror surface after 1h hyperpolishing with 17 nm CeO_2 NPs slurry.
